# Calibration Method Based on Virtual Gear Artefact for Computer Vision Measuring Instrument of Fine Pitch Gear

**DOI:** 10.3390/s24072289

**Published:** 2024-04-03

**Authors:** Xiaoyi Wang, Tianyang Yao, Zhaoyao Shi

**Affiliations:** 1 School of Mechatornics Engineering, Henan University of Science and Technology, Luoyang 471003, China; yty0101@139.com; 2 Henan Key Laboratory of Mechanical Design and Transmission System, Henan University of Science and Technology, Luoyang 471003, China; 3 College of Mechanical & Energy Engineering, Beijing University of Technology, Beijing 100124, China; shizhaoyao@bjut.edu.cn

**Keywords:** gear, vision measuring, master gear, virtual gear artefact

## Abstract

The verification of the correctness, adaptability, and robustness of software systems in modern precision measurement instruments is of great significance. Due to the difficulty in processing and calibrating high-precision fine-pitch gear artefacts, the function verification and accuracy calibration of vision measurement instruments for the fine-pitch gear have become a challenge. The calibration method of the gear vision measurement system based on the virtual gear artefact involves two steps, namely obtaining and applying the virtual artefact. The obtained virtual gear artefact has the same geometric features, error features, and image edge features as the real artefact. The calibration method based on the virtual artefact can complete the correctness verification of the gear vision measurement system, and is superior to the traditional methods in adaptability verification, robustness verification, and fault analysis. In a test, the characteristic error of the virtual gear artefact could be reproduced with the original shape in the evaluation results of the computer vision gear measurement (CVGM) system, while the reproduction error did not exceed 1.9 μm. This can meet the requirements of the verification of the gear vision measurement software. The application of the virtual gear artefact can significantly improve the accuracy and robustness of the computer vision measuring instrument of the fine-pitch gear.

## 1. Introduction

Fine-pitch gears are gears with a module less than 1 mm. Fine-pitch gears are both the core parts of major equipment and the basic parts of household products, and are widely used in automotive, aerospace, instrumentation, and other industries [1]. Measurement is an important means to analyze the sources of gear process errors and predict the quality of gear products [2,3,4,5,6]. Although domestic and foreign instrument companies have developed various precision measuring equipment that can be used for fine-pitch gears, the field of fine-pitch gear measurement still faces problems such as high measurement difficulty, low accuracy, low efficiency, and expensive instrument prices due to the small geometric size, high accuracy requirements, poor rigidity, and easy deformation of the fine-pitch gear. Especially for gears with a module below 0.3 mm, how to achieve high-precision, low-cost, and high-efficiency measurement has been a long-standing challenge that remains unresolved.

At present, there are two common types of fine-pitch gear measurement equipment on the market: gear measurement centers based on contact probe and image measurement equipment based on visual measurement. Compared with contact measurement, computer vision measurement has better accuracy, higher efficiency, lower measurement force, and can measure tiny structures, forming better application prospects than contact measurement in the field of fine-pitch gear measurement [7,8,9,10]. In recent years, various measuring instruments based on computer vision technology have emerged, including video measuring machines, flash measuring machines, and online visual sorting machines, that can be applied to fine-pitch gears. Among them, the CVGM (computer vision gear measurement) measuring instrument has been specially developed for visual measurement of fine-pitch gears, featuring automatic identification of gear parameters, high measurement efficiency, rich information, and can provide an overall gear error analysis report. However, due to the lack of small module and high precision master gear artifacts, CVGM, and other visual measurement instruments for fine-pitch gears, are faced with difficulties in accuracy calibration and functional verification [11].

For gear vision measurement instruments, the correctness, adaptability, and robustness of the measurement system, especially the measurement software system, all need to be verified by using a gear artefact. Correctness refers to the ability of a gear vision measurement system to give correct measurement results when measuring gear artifacts or product gears with high precision and known errors (with calibration values). Adaptability is the ability of a gear vision measurement system to measure correctly any gear whose basic gear parameters (number of teeth, module, pressure angle, etc.) are within a reasonable range. Robustness refers to the ability of a gear vision measurement system to handle the anomalies of a measured gear such as incorrect number of teeth, incorrect module, broken teeth, significant errors, etc., in a reasonable manner.

Measurement accuracy is the most critical technical indicator of gear vision measurement systems, which usually needs to be verified through the measuring master gear. The accuracy of a visual measurement system is determined by both hardware and software factors. Hardware factors include camera (mainly pixel, resolution, and quantization noise of CCD/CMOS and other sensors), lens (resolution, and distortion), and light source (illumination uniformity, and stabilization), etc. Software factors include the correctness, adaptability, and robustness of algorithms for edge extraction, filtering, and feature recognition. For gear vision measurement software, software factors also include the correctness, adaptability, and robustness of the calculation and the evaluation algorithms for gear accuracy indicators.

With the development of modern measurement instrument technology, the role and status of software in measurement systems continue to increase, making measurement errors caused by software factors impossible to be ignored. In modern precision measuring instruments, data collection, processing, and application are the core functions completed by software algorithms. Taking gear vision measurement software as an example, the original image data of the tested part obtained by photoelectric systems such as lenses, cameras, and light sources usually need to go through processes such as filtering, edge extraction, gear basic-parameter calculation, tooth-profile data extraction, gear accuracy index calculation, accuracy evaluation, and other processes. After going through these processes, the required information in gear measurement reports can be obtained including the individual deviations such as pitch, profile, and helix, as well as radial runout, tangential comprehensive deviation, and radial comprehensive deviation. Therefore, the testing and verification of the correctness, adaptability, and robustness of software algorithms in measuring instruments are of great significance. As visual measurement systems are widely applied, there will be an increasing demand for verification of visual measurement software.

Artefact is a commonly used tool for verifying instrument measurement accuracy and functions of instruments [12]. When calibrating gear measuring instruments, involute artifacts, helical artifacts, and master gear artifacts are commonly used standards. By using calibrated instruments to measure and evaluate the artefact, and by comparing the measured values of the artefact with the nominal values of the indicator parameters in the verification certificate of that artefact (measured by measuring instruments with higher precision), the correctness of the measurement system can be verified.

Physical artifacts used for precision measuring instrument verification usually have high manufacturing accuracy and need to be verified by instruments with higher accuracy levels to obtain the nominal values of the artefact parameters. In order to reduce the difficulty of processing, the working surface of the artefact should be designed as a simple geometric shape that can help achieve precision machining, such as a flat, cylindrical, or spherical shape. However, artifacts composed of simple geometric shapes cannot fully realize the calibration function of complex surface measuring instruments. Therefore, artifacts with high-precision complex surfaces are usually essential. In the field of gear measurement, gear measurement centers usually require gear artifacts with involute helical surfaces in the calibration. Therefore, high-precision gear artifacts have always been one of the research focuses in the field of gear measurement and have provided a lot of research results [13].

However, in fine-pitch gear measurement, especially for fine-pitch gears with a module less than 0.3 mm, obtaining high-precision gear artifacts has always been a challenge [14,15]. This is mainly due to two reasons: First, fine-pitch gears have complex geometric shapes and small dimensions, making it difficult to produce high-precision gear artifacts; second, there is a lack of ultra-precision measuring instruments for fine-pitch gears with high-precision micro-force measuring probes. What is more, even if master gears can be processed to meet the accuracy requirements, it is difficult to achieve the calibration measurements [16,17,18]. Therefore, verification of software and hardware functions of the fine-pitch gear measurement system and calibration of the instrument are both challenges to be solved. At present, fine-pitch gear measuring instruments can only evaluate the repeatability of the measurement results by repeatedly measuring the same gear. However, the correctness of the measurement results is difficult to verify, and the correctness of the measurement software algorithm itself and its implementation process, as well as whether calculation results meet correctness and accuracy requirements, cannot be verified. Moreover, it is impossible to achieve the transmission and traceability of measurement values for fine-pitch gears.

In fact, there are also shortcomings in the calibration of physical artifact application in modern measuring instruments that combine software and hardware. Taking visual measurement systems as an example, when users use physical artifacts for system calibration, they often cannot accurately locate whether the measurement error comes from the software parts (such as algorithms) or the hardware parts (such as lenses, cameras, light sources, etc.). Zhang et al. [19,20] used simulated images for wheat breeding selection in their study on generating an adversarial-driven, cross-aware network for hyperspectral wheat variety identification, indicating that using images with specific features generated by algorithms to calibrate measurement software may become a better method.

This paper proposes a calibration method for a gear vision measurement system based on the virtual gear artefact, which includes two steps, namely obtaining and applying the virtual artefact. The obtainment of the virtual gear artefact includes three steps: establishing mathematical models, superimposing gear error features, and superimposing image features. The obtained virtual gear artefact has the same geometric features, error features, and image edge features as the real artefact. The error of the virtual gear artefact is known and can be applied to calibrate the gear visual measurement system. Calibration of the gear vision measurement system can be achieved by measuring the virtual gear artefact with the gear vision measurement system and comparing the measurement results.

The calibration method based on the virtual artefact can complete the correctness verification of the gear vision measurement system. Compared to existing methods, virtual gear artifacts have the advantages of low cost, high accuracy, and easy access. So, they can be used instead of physical artifacts, which are practically difficult to obtain, to verify the correctness, adaptability, and robustness of the gear vision measurement system, especially to verify that of the measurement software algorithm. They can also distinguish between software and hardware problems in gear vision measurement systems. This method was applied in the CVGM measurement system and achieved good results, which could significantly improve the detection accuracy and robustness of visual measurement software.

The contributions of this paper are summarized as follows:

1. This paper propose a method for calibrating gear vision measuring instruments with virtual gear artefacts
2. The method of acquiring a virtual gear artefact is proposed, including the generation method of geometric features, error features, and image edge features
3. The feasibility of using a virtual artefact to verify gear vision measurement software is verified through experiment.

## 2. Acquisition of Virtual Gear Artefact

The virtual gear artefact that can be used for gear visual measurement software calibration is in fact a gear image with no errors or known errors, having the same or similar features as the actual measured images of the actual artefact. In order to obtain a virtual gear artefact that meets functional requirements, it is necessary to consider multiple key elements based on gear features and image features, and design them in combination with hardware parameters of the visual measurement system. Gear features can be divided into error-free gear geometric features and gear error features to be superimposed. Image features include pixel, resolution, and contour edge features.

### 2.1. Geometric Features of Gears

Since the mathematician L. Euler introduced involute tooth profiles in 1765, with the invention of the gear roll processing method in the late 19th century, and gear technology progress made by the development of the automobile industry in the early 20th century, more than 90% of current gear transmission systems use involute gears due to their good transmission performance and low processing cost. Influenced by the limitations of existing technology, in most cases gear vision measurement technology based on non-contact image measurement principle can only be used in spur cylindrical gear measurement, but not in spiral gear and bevel gear measurement. Therefore, this paper takes the most common involute spur gear as an example to illustrate the geometric characteristics of the virtual gear artefact.

The profile of each tooth on an error-free gear is identical, and the shape of the left and right tooth surfaces of the same tooth is symmetrical. Thus, by establishing a parameter equation for only one side of the tooth surface, all profile equations can be derived through symmetry and periodicity. There are various methods for establishing an involute tooth-profile equation for selection [21,22].

**Figure 1:**
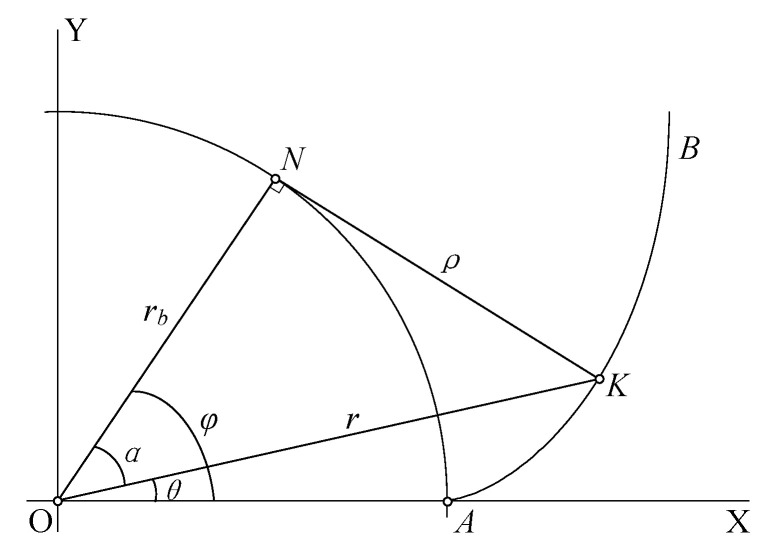
Calculation of involute profile of single right flank.

As shown in Figure 1, *r_b_* is the radius of the base circle, with the center of the base circle as the origin O, and the ray passing through the starting point *A* of the right tooth involute tooth profile on the base circle as *X*-axis positive, to establish a right-handed coordinate system O-XY. In Figure 1, *K* is any point on the tooth surface with a radius of *r*, and *N* is the tangent point between the normal of the tooth surface at point *K* and the base circle. The angle *α* is the pressure angle of the tooth surface at point *K*, *θ* is the roll angle of the involute, the involute angle of involute is *φ*, and the length of the line segment *KN* is the corresponding roll length of the involute at point *K*.

The involute function is defined as follows:

(1) $$inv\left(\alpha \right)=\tan\left(\alpha \right)-\alpha$$

It can also be written as below:

(2) $$\theta =inv\left(\alpha \right)$$

The roll arc length *ρ* corresponding to the point (i.e., *K*) with a pressure angle of *α* on the tooth profile is denoted as follows:

(3) $$\rho \left(\alpha \right)=\left|\bar{\mathrm{KN}}\right|=r_{b}/\tan\left(\alpha \right)$$

Therefore, in the coordinate system O-XY, the involute tooth-profile equation of the right tooth surface of an involute spur cylindrical gear can be expressed as below:

(4) $$\left\{\begin{array}{l} x_{K}\left(\alpha \right)=\rho \left(\alpha \right)\cdot \cos\left(inv\left(\alpha \right)\right)\\ y_{K}\left(\alpha \right)=\rho \left(\alpha \right)\cdot \sin\left(inv\left(\alpha \right)\right) \end{array}\right.$$

The horizontal coordinate of point *K* on the involute in the O-XY coordinate system is *x_k_*, and the vertical coordinate is *y_k_*. When α ∈ (0,α_tα_], Equation (4) is the same as the curve of the involute tooth profile from the base circle to the tooth-tip circle; where α_tα_ is the pressure angle of the tooth-tip end face. The tip circle is denoted as *r_a_*, it can be represented by the following formula:

(5) $$\alpha_{ta}=\arccos\left(r_{b}/r_{a}\right)$$

Equation (4) provides an error-free calculation method for the involute tooth profile of the virtual artefact. Based on the parameters of the virtual master gear to be simulated, tooth-top arc, tooth-root arc, and tooth-root transition curve can be calculated. Than a complete virtual master gear profile can be obtained.

### 2.2. Gear Error Characteristics

The use of error-free gear artifacts can only verify whether the measurement system works properly under suboptimal conditions, but cannot verify the correctness and effectiveness of the measurement system when there are various forms of errors in the measured gear. Therefore, it is necessary to develop gear-shape physical artifacts with characteristic errors. Similarly, in order to fully verify the correctness and effectiveness of the measurement system under actual working conditions, it is necessary to develop virtual master gear artifacts with various forms of error characteristics.

According to the causes of errors, gear machining errors and instrument measurement errors can be divided into three categories: systematic errors, random errors, and thick errors. When designing virtual gear artifacts, these three types of errors should also be superimposed as needed.

#### 2.2.1. Systematic Error

There are two simulation methods that can generate virtual master artifacts with systematic errors. One is to simulate the geometric shape errors of gears based on the principle of actual hobbing or grinding process errors. Another approach is to directly superimpose any form of design error on the error-free gear profile, ignoring the causes of the error. The virtual artefact designed according to the former method can be used for simulation and verification of the gear process-error analysis function based on gear measurement, while the virtual artefact designed according to the latter method is mainly used for simulation and verification of measurement and the evaluation function of the gear visual measurement system.

The common sources of process errors in actual gear machining are the installation error of the gear blank, machine tool transmission chain error, gear spindle runout error, hob spindle runout error, tool installation error, tool wear or tool manufacturing error, etc. Manifestations of errors from various sources in gear products include pitch deviation, profile deviation, pressure-angle deviation, tooth-thickness deviation, radial comprehensive deviation, tangential comprehensive deviation, etc. These specific process errors can be analyzed and calculated based on the geometrical error model of gear hobbing [23].

The method of directly superimposing any known error to the tooth profile of an error-free gear is relatively simple, while this method can meet the verification of the correctness and adaptability of the virtual master gear artefact for the gear visual measurement system. The magnitude and frequency of the error superimposed on the simulated gear should be set based on the actual gear-error situation and the verified instrument ability. The error superimposed on the virtual master gear artefact can be reproduced on the gear deviation curve obtained after this virtual master gear has been correctly measured and analyzed.

Common gear measuring instruments, like gear measuring centers, draw profile deviation curves by referring to ISO-1328.1-2013 [24] and other international standards for gear accuracy. These profile deviation curves are usually drawn with the corresponding roll length of each point on the involute tooth profile as independent variable. In order to observe whether the profile deviation curve obtained from the measurement of the virtual gear artefact with errors is the same as the shape of the superimposed known error curve, the known error curve to be superimposed should also be defined with the roll length as the independent variable.

**Figure 2:**
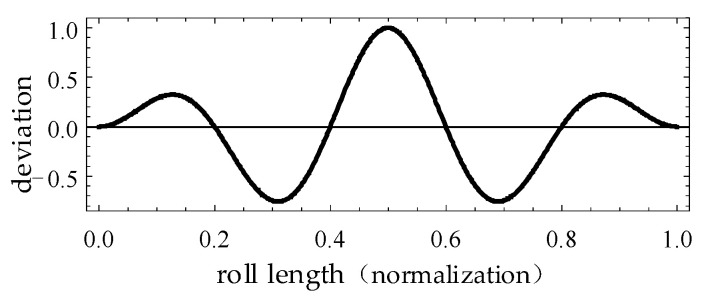
Example of a characteristic error curve superimposed on profile of virtual artefact.

Figure 2 is a characteristic error curve that can be superimposed on the tooth profile of a virtual gear artefact. The formula for this characteristic error curve is as follows:

(6) $$K\left(\rho \right)=\left(1-2\cdot {\left|\frac{\rho }{\rho_{a}}-\frac{1}{2}\right|}^{1.5}\right)\cdot \sin\left(\frac{5\pi \rho }{\rho_{a}}\right)$$

In Equation (6), the roll length is represented by *ρ*, the roll length at the top of the virtual master gear tooth is represented by *ρ_α_*, and the calculation formula is as follows:

(7) $$\rho_{a}=r_{b}\tan\left(\alpha_{ta}\right)$$

This error curve is left–right symmetric, and the horizontal coordinate is the normalized roll length *ρ*, which takes values in the range [0, 1] and corresponds to the involute tooth profile between the base circle and the tip circle. The vertical axis represents the coefficient *K* of the deviation to be superimposed on the tooth profile. The maximum value point of the deviation coefficient *K* is (0.5, 1), the minimum value points are (0.306, −0.825) and (0.694, −0.825), and the peak valley value (also known as PV value, i.e., peak valley value) is 1.825. When superimposing the characteristic error curve on the tooth profile of the virtual master gear, it is necessary to determine the magnitude of the error to be superimposed. The maximum magnitude of the error curve is usually taken as 0.1 times the module m of the virtual master gear. At this point, the total profile deviation *F_a_* should theoretically be 0.1825 times the module m.

In order to superimpose the above errors on the virtual master gear, it is necessary to modify the parameter equation of the gear to a parameter equation with the extension length corresponding to the tooth-profile point as the independent variable. In the coordinate system O-XY shown in Figure 1, the parametric equations for the involute tooth profile with the roll length *ρ* as the independent variable are established as follows:

(8) $$\left\{\begin{array}{l} x_{K}\left(\rho \right)=r_{b}\cdot \cos\left(\varphi \right)+\rho \cdot \sin\left(\varphi \right)\\ y_{K}\left(\rho \right)=r_{b}\cdot \sin\left(\varphi \right)+\rho \cdot \cos\left(\varphi \right) \end{array}\right.$$

The angle *φ* in Equation (8) must also be presented as a function of the roll *ρ*, that is the following:

(9) $$\varphi \left(\rho \right)=a\tan\left(\rho /r_{b}\right)+\mathrm{inv}\left(a\tan\left(\rho /r_{b}\right)\right)$$

When *ρ* ∈ (0, *ρ_α_*], Equation (8) corresponds to the involute tooth-profile part from the base circle to the tooth-tip circle.

When adding errors to the involute tooth profile in Equation (8), according to gear accuracy evaluation standards (such as ISO-1328), the errors are added along the normal direction of the tooth surface. In Figure 1, the parameter equation for the tangent point *N* of the base circle corresponding to the involute tooth-profile point *K* with the roll length *ρ* as the independent variable is the following:

(10) $$\left\{\begin{array}{l} x_{N}\left(\rho \right)=r_{b}\cdot \cos\left(\varphi \right)\\ y_{N}\left(\rho \right)=r_{b}\cdot \sin\left(\varphi \right) \end{array}\right.$$

The angle *φ* in Equation (10) is also calculated according to Equation (9).

The normal direction at point *K* of the involute tooth profile can be calculated from the coordinates of point *N* and point *K*, and then the tooth profile of the virtual gear artefact can be obtained after adding systematic errors.

#### 2.2.2. Random Error

The reason why random errors are added on the virtual gear artefact is mainly to verify the sensitivity of the measurement system software algorithm to small differences in the gear profile, and secondly to verify the robustness of the measurement system software algorithm. If excessive filtering is added to this measuring software algorithm, the ability to perceive small tooth-profile differences may be lost while obtaining stable measurement results. The magnitude of the superimposed random error should be limited, usually to lower than one-third of the maximum magnitude of the system error mentioned above, and greater than the minimum resolution of the verified measurement system. It should be noted that random errors superimposed on the virtual gear artefact are “pseudo-random”, and these random errors are recorded and used as known data for the virtual artefact.

Similar to the method of adding systematic errors, by adding a random number term with limited magnitude to the right of Formula (6), random errors can be added on the theoretical tooth profile of the virtual gear artefact.

#### 2.2.3. Thick Error

The reason why thick errors are superimposed on the virtual gear artefact is mainly to verify the robustness of the measurement system software, that is, the ability of the system to provide reasonable measurement results in abnormal situations. Thick errors that can be superimposed on the virtual gear artefact include incorrect number of teeth, incorrect module, broken teeth, adjacent teeth, no inner hole, large shift, end reflection, as well as an abnormally large magnitude profile deviation, pitch deviation, tip circle diameter deviation, etc.

Newly developed measurement systems often stop operating due to abnormalities in the early stages of use. Traditionally, this situation can only be curbed by experienced developers, but such abnormal downtime cannot be completely avoided. Before the measurement system is put into use, specific tests on various known abnormal situations can be conducted by reasonably using virtual artifacts with superimposed thick errors. It not only greatly improves the robustness of the newly developed gear vision measurement system, but also saves the cost of designing and manufacturing a large number of physical gears, thick with specific errors.

### 2.3. Image Features

The image features of the virtual gear artefact should be designed in conjunction with the key parameters of the visual measurement system to be verified, such as pixel, resolution, lens magnification, while they should also be in line with the edge features of the actual images captured by the visual measurement system to be verified.

Gear vision measurement systems usually obtain the profile of the measured gear through edge extraction algorithms, and then calculate and evaluate the gear error. Therefore, the virtual artefact images generated by simulation should have as close an edge features as possible to the images captured by the actual visual measurement system. These edge features mainly include the following: (1) gray on both sides of the edge, (2) width of the edge (usually measured in pixels), (3) gray gradient regularity of the edge, and (4) the distribution artefact of the random error of gray.

In this case, the gray shades on both sides of the edge are mainly determined by illumination brightness and camera gain: edge width refers to the number of pixels occupied by blurred edges whose grayscale values gradually change from the inside to the outside of the entity; the gradient variation rule of pixel gray within the edge range is related to factors such as actual camera, lens, and light source used, and is a key factor affecting the accuracy of sub-pixel edge extraction; due to changes in gain, quantization error, and brightness of the light source of the camera’s photosensitive components, there may be random fluctuations in the gray of each point in the actual captured image, which can also affect the accuracy of the visual measurement system.

When generating virtual master gear images, the pixel gray and random error distribution patterns of the non-edge parts on both sides of the edge can be directly extracted from the actual measured image or specified based on experience.

In order to improve measurement accuracy, gear vision measurement instruments usually use sub-pixel edge extraction algorithms. From a practical perspective, the edge width and edge gray gradient regularity of the virtual gear artefact image have a significant influence on the sub-pixel algorithm. Therefore, when verifying the measurement software, the width of the blurred edge area should be set to be consistent with the edge width in the actual measured image. Gray gradient regularity inside the blurred edge area can usually be set according to the arctangent function regularity. To achieve better results, the gray gradient regularity of the image edges in the actual measured image simulation can help [25,26].

## 3. Application of Virtual Gear Artefact

The application methods of the virtual gear artefact and physical artefact have both similarities and their own characteristics.

Similar to the physical master gear artefact, the virtual artefact can be used to verify the correctness of the visual gear measurement system. The basic method is to input the simulated virtual gear artefact image into the visual measurement system software, and obtain the corresponding measurement results. Then the known errors when generating the virtual artefact can be compared with the measured errors obtained from measuring the virtual artefact to evaluate the correctness and accuracy of the measurement system software.

Unlike the physical artefact, the virtual master gear artefact can only verify the correctness of the visual measurement software algorithms, but cannot directly verify whether the optical system (lenses, and light sources), camera system and other hardware systems are working normally. However, this feature instead brings a unique effect, that is, the virtual artefact can achieve the independent testing of the software functions of the measurement system, while the testing process and results are not limited by the actual hardware conditions such as cameras and lenses. When the actual visual gear measurement system experiences “soft faults” such as repeated measurement results, poor accuracy, or irregular downtime during use, the virtual gear artefact can be used to distinguish the root cause of the problem. During the development process, this feature is beneficial for analyzing the root causes of the abnormal measurement results, which can accelerate the progress of the measurement software optimization work.

The adaptability of gear vision measurement systems is the ability to handle various conditions in which different tooth numbers, modules, errors sizes, and errors form from different combinations. In order to verify the adaptability, it is usually necessary to design and manufacture a series of gear artifacts with specified errors for verifying the measurement system. At this point, compared to using the physical artefact, using the virtual gear artefact has obvious advantages, which can greatly save costs and improve the efficiency of system development and testing.

In order to verify the robustness of the gear vision measurement system, it is usually necessary for the testing personal to add various abnormal situations on the tested gear and conduct measurement tests under the arrangement and combination of various abnormal situations. In fact, it is easier to locate and troubleshoot the problems caused by abnormalities in hardware parts such as cameras, lenses, and light sources in modern gear vision measurement systems; most robustness problems that are difficult to locate and eliminate are caused by software components. However, virtual gear artefacts can be used to complete validation experiments for these problems. From a practical perspective, the introduction of the virtual gear artefact for verification has significantly improved the robustness of the CVGM series gear visual measurement system.

In addition, during the design and development stages of visual measurement instruments, when evaluating the measurement accuracy with different lenses and camera combinations, different edge widths can be specified on the virtual artefact for simulation testing. That is, software performance testing can be carried out under the hardware schemes of different measurement systems by using virtual gear artifacts. This is a unique advantage of using the virtual artefact method for visual measurement system validation.

## 4. Testing and Analysis

**Figure 3:**
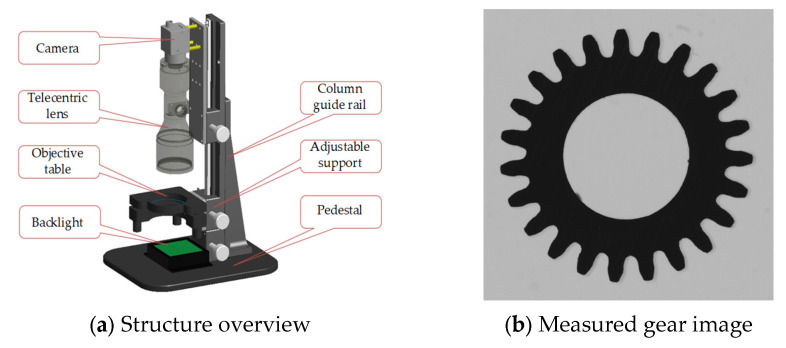
CVGM structure and gear image measured.

The verification method based on virtual gear artefact was experimentally verified in the development and application of the fine-pitch gear visual measurement system CVGM. The resolution of CMOS, pixel size of CMOS, lens magnification, and other parameters listed in the table are important hardware factors that affect the evaluation results of the measuring instruments. Table 1 shows the main technical parameters of the CVGM-H-12M system used for the test. Figure 3 shows the schematic diagram of the CVGM-H-12M measurement system and the gear images collected by the system. The CVGM-H-12M system uses a flat backlight for illumination and places the measured gear on the object table. The object table has a horizontal accommodation adjustment function, which can adjust the axis of the tested gear to be parallel to the optical axis of the telecentric lens. The software of this instrument has the function of inputting, analyzing, and evaluating the images of the gears. Virtual gear artifacts can be used to verify and test the correctness, adaptability, and robustness of the software algorithms.

The range of pixel gray values measured by the CVGM-H-12M system is 0–255. Under appropriate lighting brightness, the gray of the foreground (darker) in the collected image is usually ≤10, and the gray of the background (brighter) is usually ≥70.

Targeted experiments were conducted on the acquisition and application of the virtual gear artefact based on the hardware parameters and image features of the CVGM measurement system. The pixel size of the virtual artefact image generated in the test is 3.7 microns, and features such as gray on both sides of the edge and edge width are also set according to the regularity of the actual image measured by the instrument.

**Figure 4:**
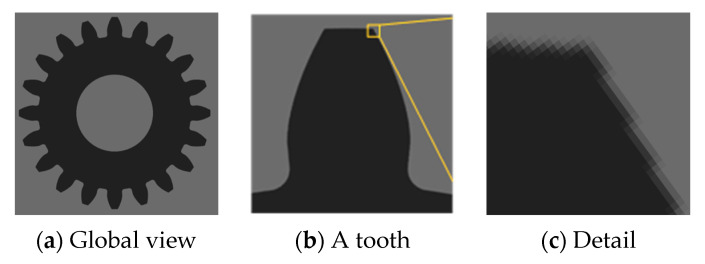
Virtual gear artefact without error.

Figure 4 shows the global view, tooth, and local detail images (with an edge width of 3 pixels) of the error-free virtual gear artefact generated according to Formula (4).

In order to show the measurement results and to conduct the manual judgment of tooth-shape symmetry, CVGM measurement software can rotate any placed gear image to the vertical upward direction of the gear teeth. Unlike the common images captured directly by camera, the gear teeth image in Figure 4 is rotated, so the pixel edges are not along the horizontal and vertical directions, but tilted.

**Figure 5:**
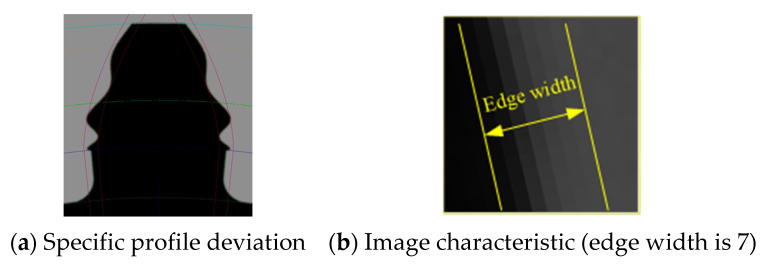
Virtual gear artefact with designed error.

Figure 5a shows the local profile of a virtual gear artefact with the error superimposed on the involute tooth profile, where the profile error regularity is the same as that of Equation (6). To highlight the error feature, the maximum magnitude of the error is set to 0.1 times the module. To form a contrast, the shape of the ideal involute is drawn with pink thin lines in the graph. Among them, Figure 4 and Figure 5 are mainly used to verify the correctness and accuracy of the gear vision measurement system. The profile-error shape in Figure 5a is not exactly the same as the curve shape in Figure 2. The main reason is that the abscissa of the curve in Figure 2 is the roll length, and the relationship between the roll length and the gear diameter is non-linear.

Figure 5b shows the local profile of the virtual artefact in Figure 5a, with an edge width of seven pixels. The gray variation regularities of the image edges captured by the measurement systems with different hardware compositions when measuring the physical gears are different. Here the inverse tangent function curve along the edge of the normal direction is used as the gray gradient change regularity, which is a more universal edge gray-scale change regularity.

**Figure 6:**
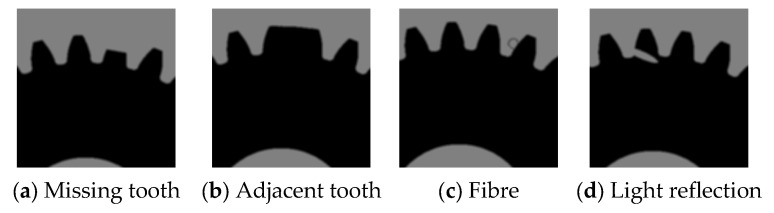
Virtual gear artefact with abnormalities.

Figure 6 shows the virtual gear artefact images with typical anomalies (missing tooth, adjacent teeth, fibers, and reflections). These virtual artifacts can be used to verify the robustness of the actual gear vision measurement instruments. The black circular anomaly caused by fiber dirt at the edge of the profile in Figure 6c once caused an error in a certain version of the measurement software, because the edge profile extracted from the image would self-cross. Using virtual gear artifacts can fix various abnormal phenomena of the tested gear, which can be used for verification testing of subsequent versions of software to avoid the same faults from occurring again.

**Figure 7:**
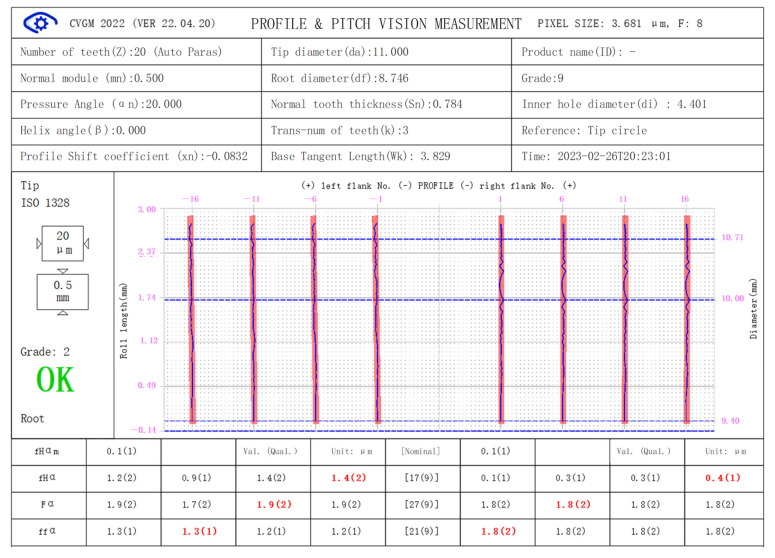
Measurements of error-free virtual artefact.

**Figure 8:**
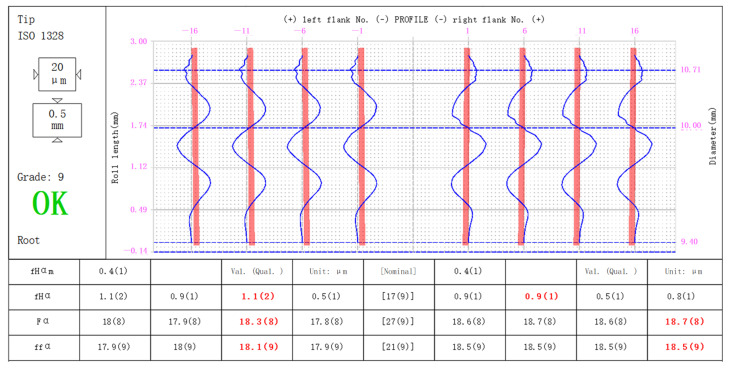
Measurements of virtual artefact with specific error.

Figure 7 and Figure 8 show the measurement results of the error-free virtual gear artefact generated according to Equation (4) and the virtual gear artefact with specific errors generated according to Equation (6) (PV value is 18.25 μM) by using micro-gear visual measurement software (CVGM 2022).

In Figure 7, the maximum total profile deviation of the error-free virtual gear artefact is 1.9 microns, which is caused by the quantization error of the theoretical profile when converted to gear pixels and the error of the edge extraction algorithm. This is the maximum accuracy that this version of the software algorithm can achieve in the optical measurement system of the instrument. According to the gear accuracy standard (ISO-1328.1:2013), the overall accuracy index of this virtual gear artefact is rated as class 2. The tolerance ratio of adjacent accuracy classes in the gear accuracy standard is approximately 1.414. According to the principle that the measurement error is one third of the measured gear tolerance, this measurement system can meet 5–6 of accuracy. To further improve measurement accuracy, it is necessary to take measures such as improving the resolution of the lens, reducing the pixel size in the image (approximately equal to the product of the actual pixel size on the photosensitive component multiplied by the lens magnification), or making new progress in the sub-pixel edge extraction algorithm of the measurement software.

The basic gear parameters of the virtual gear artefact in Figure 8 are the same as those given in Figure 7. The profile deviation curve measured in Figure 8 has the same extreme values, trends, and shapes as the known error curve superimposed on the virtual gear artefact shown in Figure 2. In the experiment, the total profile deviation *F_a_* superimposed on the generated virtual artefact was 18.25 μm. The maximum *F_a_* in the measurement results is 18.7 μm, the minimum is 17.8 μm. The indication error is ≤ 0.45 μm.

**Figure 9:**
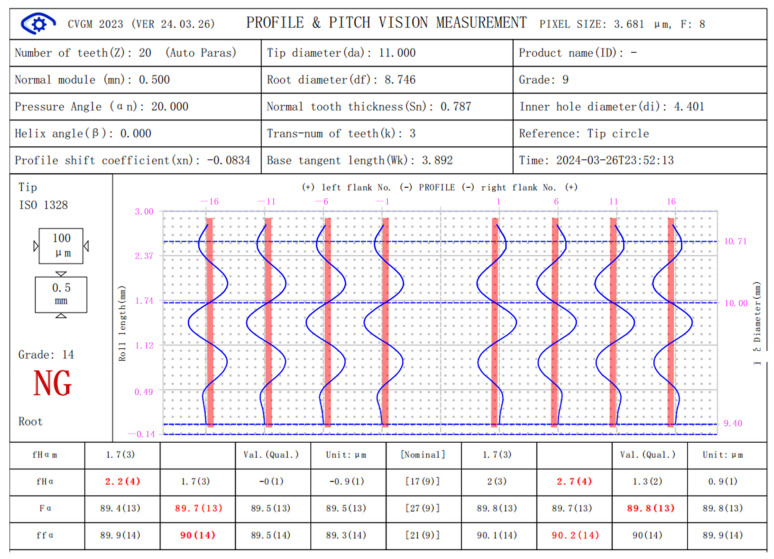
Measurements of virtual artefact with a large amplitude profile deviation.

**Figure 10:**
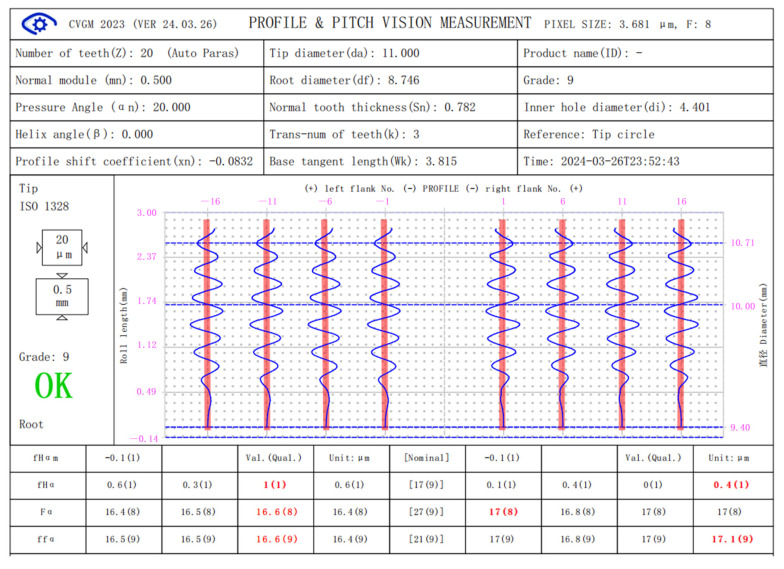
Measurements of virtual artefact with a high-frequency profile deviation.

By modifying the parameters in Equation (6), it is possible to obtain error curves with different amplitudes and frequencies, and thus obtain virtual artifacts with different errors superimposed. Figure 9 shows the measurement results of a virtual artifact with a large amplitude profile deviation superimposed. Figure 10 shows the measurement results of a virtual artifact with a high-frequency profile deviation superimposed.

The measurement results indicate that the virtual master gear generation algorithm and the verification test based on this virtual artefact are correct and feasible.

## 5. Conclusions

Due to the difficulties in manufacturing and calibration of the physical artifacts for small module gears, visual measuring instruments for small module gears with a module less than 0.3 mm cannot be calibrated. To address this issue, this paper proposed a verification method for small module gear visual measurement systems based on virtual master gear artifacts. The necessary gear geometric features, gear error features, and gear image features of virtual master gear artifacts are provided, and the application methods of virtual master gear artifacts in verifying the correctness, adaptability, and robustness of small module gear visual measurement systems are summarized.

In the experiment, when the pixel size of the error-free virtual artefact image is 3.7 microns, the overall accuracy rating given by the CVGM system is class 2. The measurement results show that the measurement system can meet the gear measurement requirements of 5–6 levels of accuracy. The difference between the measured and theoretical values of the total profile deviation *F_a_* of a virtual artefact with errors is ≤0.45 μm. The measurement results indicate that the algorithm for generating virtual gear artifacts proposed in this paper is feasible, and the validation testing of the virtual artifacts generated based on this algorithm is also feasible.

Theoretical analysis and experimental data show that the virtual gear artefact can be used for the verification of the correctness, adaptability, and robustness of the fine-pitch gear visual measurement system, can solve the problem of calibrating the function and performance indexes of the fine-pitch gear visual measurement instrument, and has the following advantages:

(1) The processing and calibration cost of the virtual gear artefact is lower than that of the physical artefact. When using the virtual gear artefact to verify the correctness of the measurement system, the true values corresponding to the various measurement indicators can be directly obtained. In addition, virtual artifacts with various characteristic errors and abnormal defects can be extensively used to verify the adaptability and robustness of the testing systems. Therefore, the virtual gear artefact is significantly better than the traditional physical artefact in verifying the correctness, adaptability, and robustness of fine-pitch gear visual measurement systems.
(2) The virtual gear artefact can effectively distinguish hardware and software factors when analyzing the sources of visual measurement errors in fine-pitch gears, which is beneficial for quickly locating the root cause of measurement errors, improving instrument measurement accuracy, and shortening the development and troubleshooting period of the instrument.
(3) During the instrument design and development phase, virtual gear artifacts with different edge features can be used for instrument performance analysis and testing under various hardware schemes, which can optimize the design scheme, reduce research and development costs, and accelerate the development progress.

Although virtual gear artifacts cannot completely replace the physical artifacts, for small or micro-module gears that are difficult or impossible to obtain physical artifacts for, introducing virtual gear artifacts in the verification of visual measurement instruments can greatly improve the accuracy, adaptability, and robustness of the measurement system. Therefore, the virtual gear artefact and its usage method have important research significance and application value.

In the future, computer vision will become an important detection method for key basic components such as gears. Virtual artefact technology represented by virtual gear artifacts proposed in this paper will play an increasingly important role in the calibration and verification process of visual measurement systems.

## Figures and Tables

**Table 1:** Main parameters of CVGM-H-12M system.

Number	Lens Parameters	Data
1	CMOS resolution	4000 × 3036
2	CMOS pixel size	1.85 µm × 1.85 µm
3	Lens magnification	0.5
4	MTF	>0.3@170 lp/mm
5	Object field of view	14.8 mm × 11.2 mm
6	Object pixel size	3.7 µm × 3.7 µm

## Data Availability

The data that support the findings of this study are available from the corresponding author upon reasonable request.

## References

[1] Shi Z., Zhang W., Qu H. (2011). Development of measuring machine based on single-flank testing for fint-pitch gears. Chin. J. Sci. Instrum..

[2] Goch G., Guenther A., Peng Y., Ni K. (2023). Gear metrology—An update. CIRP Ann. Manuf. Technol..

[3] Cacho R.A., Mazo J.S., Arteta M.P. (2013). Verification Methods for Micro Gears. Analysis of Double Flank Roll Testing Applied to Micro Gears. Procedia Eng..

[4] Ye S., Wang Z., Qu X. (2000). Review and Prospect of Precision Inspection. China Mech. Eng..

[5] Fei Y. (1998). Research and Progress in Error Theory. Metrol. Sci. Technol..

[6] Fei Y. (2000). Research Progress and Future of Several Issues in Precision Theory. China Mech. Eng..

[7] Tang J., Liu X., Li R. (2021). Vision measurement of pitch and profile deviations for small modulus gears with unknown parameters. Opt. Precis. Eng..

[8] Gadelmawla E.S. (2011). Computer vision algorithms for measurement and inspection of spur gears. Measurement.

[9] Moru D.K., Borro D. (2019). A machine vision algorithm for quality control inspection of gears. Int. J. Adv. Manuf. Technol..

[10] Zhi S., Zhao W., Zhao W., Duan Z., Sun H. (2018). Visual measurement method of pitch machine based on gear local image. Chin. J. Sci. Instrum..

[11] Kondo Y. (2009). Development of a Novel Artifact as a Reference for Gear Pitch Measuring Instruments. J. Manuf. Sci. Eng..

[12] Greif N., Schrepf H., Richter D. (2006). Software validation in metrology A case study for a GUM-supporting software. Measurement.

[13] Ling S., Ling M., Shi Z., Bo L., Wang L. (2022). Measurement comparison for class-1 gear involute artifact in China. Opt. Precis. Eng..

[14] Tu X., Xie H., Feng G., Fu Y., Huang W., Ding H. (2008). New ideas for precision measurement technology and methods for batch involute microgears. Tool Eng..

[15] Feng G., Xie H., Ye Y., Fu Y., Tu X., Ding H., Chen Z., Huang W. A New Efficient Technology for Measuring of the Fine-pitch Gear Accuracy in Batch Production. Proceedings of the 11th Annual Conference of the China Association for Science and Technology on Independent Innovation and Sustainable Growth.

[16] Shao Y., Zhang Y., Gu G., Gu J., Zan P. (2017). Research of spur gear detection method based on minimum convex hull. J. Electron. Meas. Instrum..

[17] Wang N., Duan Z., Zhao W., Du P., Duan B. (2017). Research of the Visual Measurement Method of Gear Tooth Profile Total Deviation. J. Mech. Transm..

[18] Albers A., Burkardt N., Deigendesch T., Ellmer C., Hauser S. (2008). Validation of micromechanical systems. Microsyst. Technol..

[19] Zhao W., Li C., Zhang W., Yang L., Zhuang P., Li L., Fan K., Yang H. (2023). Embedding Global Contrastive and Local Location in Self-Supervised Learning. IEEE Trans. Circuits Syst. Video Technol..

[20] Zhang W., Li Z., Li G., Zhuang P., Hou G., Zhang Q., Li C. (2024). GACNet: Generate Adversarial-Driven Cross-Aware Network for Hyperspectral Wheat Variety Identification. IEEE Trans. Geosci. Remote Sens..

[21] Wang X., Shi Z., Shu Z., Yu B. (2017). Different Point Contact Error and Correction Method in Gear Integrated Error Measurement. J. Mech. Eng..

[22] Zhang B., Lin J. (2016). Research of Tooth Profile Error Evaluation Method based on Cartesian Coordinate Measurement Method. J. Mech. Transm..

[23] Han J., Xia H., Xia L. (2014). Geometric Error Modeling and Analysis for CNC Gear Hobbing Machine. China Mech. Eng..

[24] (2013). Cylindrical Gears—ISO System of Flank Tolerance Classification—Part 1: Definitions and Allowable Values of Deviations Relevant to Flanks of Gear Teeth.

[25] Wang W. (2009). The Research on the Edge Recognition Methods and Techniques for Potential Field. Ph.D. Thesis.

[26] Duan Z., Wang N., Fu J., Zhao W., Duan B., Zhao J. (2018). High Precision Edge Detection Algorithm for Mechanical Parts. Meas. Sci. Rev..

